# Globalization, Work, and Health: A Nordic Perspective

**DOI:** 10.3390/ijerph17207661

**Published:** 2020-10-21

**Authors:** Steffen Torp, Jon Reiersen

**Affiliations:** 1 Department of Health, Social and Welfare Studies, University of South-Eastern Norway, N-3199 Borre, Norway; 2 School of Business, University of South-Eastern Norway, N-3199 Borre, Norway; Jon.Reiersen@usn.no

**Keywords:** health promotion, workplace, labor relations, collective bargaining, unionization, globalization, precarious work, equity, empowerment

## Abstract

The Nordic countries are among the world’s leading countries in international rankings on prosperity, productivity, social equity, trust, and health. Such positive results may be linked to how these countries have organized their working life. The aim of this article is to describe core elements of the Nordic working life model (emphasizing Norway) and discuss how globalization may challenge the model, and thereby influence public health. Based on an extensive review of relevant research, we show that the Nordic working life model with a coordinated wage bargaining system between well-organized employers and employees results in productive enterprises, small wage differences, good working environments, and a high level of well-being. Global trends of liberalization of working life, increased labor migration, the platform economy, reduced unionization, and more precarious work challenge the Nordic working life model and its reliance on standard working contracts. Such a trend may result in increased inequity, reduced generalized trust, and poorer public health. Politicians and other stakeholders in the Nordic countries should cope appropriately with globalization and technological changes so that the Nordic countries will uphold their well-organized working life and good societal achievements.

## 1. Introduction

Work and employment are key activities for individuals and society. For the individual, participating in the labor market means access to income, and work also provides access to several other important benefits such as social recognition, belonging and identity, the development of personal skills, and personal control. Under the right conditions, these factors contribute positively to individual health and well-being.

The labor market and the nature of work are rapidly changing around the world. Major drivers behind this change are economic globalization and new technology. Economic globalization can be defined as widespread cross-border movement of people, capital, goods and services, and is intimately related to new technology. The revolution in information and communication technology and other technological innovation, including reduced transport costs, have made interaction between individuals, companies, and countries easier. As globalization increases, so does international competition, which in turn stimulates technological development and further globalization. The concept of globalization is broad and may include several dimensions other than the economic one, such as the spread of a specific type of culture or policy. This article, however, is largely limited to the economic dimension of globalization. Although economic globalization is nothing new, it represents a far stronger force, and with a stronger reach, than ever before.

Increased social and economic interaction between an ever-increasing group of people has been an important driver for economic development and well-being throughout history. As the markets expand, so do the opportunities to reap the benefits of trade, the division of labor, and specialization. Although globalization and new technology offer potential progress, they cannot guarantee its emergence. For example, the benefits from globalization are not guaranteed to be widely and fairly shared [1]. Some commentators even claim that globalization and new technology constrain the policies that countries can carry out to secure broad social progress [2,3,4].

The way the Nordic countries have organized their welfare systems and labor markets has produced societies with good economic performance combined with relatively low income inequalities, universal social welfare policies, and high social protection [5,6,7,8,9]. It is also well documented that these factors are vital for public health and well-being [10,11]. Although the Nordic societal model has delivered good results along many dimensions, many observers question whether the model is robust in the face of globalization and the rapid increase in cross-border movement of goods and services, capital, and people [3,4,12]. Mechanisms discussed in the literature include immigration potentially leading to reduced public support for welfare state redistribution policies, a race to the bottom in taxation creating difficulty in funding a comprehensive welfare state, and increased internationalization of the labor market putting job security and working conditions under pressure.

The relationship between a comprehensive and universal welfare state (such as in the Nordic countries) and public health is well acknowledged [10]. The welfare state plays a key role in providing social protection, health care, housing, income insurance, education, and so on—factors that are important in producing good health. An important but sometimes overlooked aspect of the Nordic model is the labor market and how it is structured in the Nordic countries. For example, the Nordic model of labor relations has played a key role in creating small wage differences and holding unemployment low. It has also fostered strong collaboration between employers and employees at both the national and the company levels. The level of conflict is low, and Nordic workers generally report a high degree of involvement in decision-making processes at the company level. In this sense, the Nordic model for working relations can be regarded as a part of a larger system for democratic innovation. Collective agreements have established formal and informal structures that give workers room to take initiative, to use their own problem-solving abilities, and to participate actively in innovation and restructuring processes [13]. All these elements contribute to a sound working environment, which again is strongly associated with good occupational and public health, in addition to high productivity and wealth creation.

By using Norway as a Nordic case, this article addresses the following questions: (1) What are the core elements of the Nordic working life model and how do they affect the nature of work and occupational health? (2) How are globalization and technological development challenging the Nordic working life model? (3) How might globalization and new technology influence occupational and public health if they are not appropriately coped with?

## 2. Methods

The current study is not a traditional empirical study nor a systematic review. In Grant and Booth’s [14] typology, it resembles a literature review. Using an interdisciplinary perspective (inspired by comparative political economy, labor relations studies, organization theory, and public health perspectives), we provide a conceptual basis for understanding key elements of the Nordic and Norwegian working life model, how this model affects work-related health and democratic innovation, and how this model may be threatened in the future. Although our study is interdisciplinary, it is embedded in a traditional social science tradition where we use several sources, ranging from available economic, social, and public health statistics, and recent empirical and theoretical research addressing possible threats against the Norwegian working life model, to support our own primary analysis and interpretation.

In our search for relevant literature on labor relations in Norway published in the past twenty years, we searched in international research databases (Proquest and Web of Science), the Norwegian University Libraries’ database Oria, and Google (for reports/gray literature) with search terms such as “Nordic Model”, “income inequality”, “labor relations”, and “health” combined with “Norway”. We searched the reference lists of already well-known publications on the current theme and of relevant publications we found through searches. Due to little international research on recent changes in the Norwegian labor market, we searched particularly for relevant “gray” reports from Norwegian research institutes. We used both Norwegian and English search terms.

We use our conceptual framework to discuss what impact recent societal changes may have on equity, well-being, and public health in the Nordic countries. Based on this discussion, we make propositions for necessary future research relevant for democratic innovations.

Finally, it should be said that this is by no means an all-embracing analysis of the Norwegian working life model. Rather, it is a novel attempt to unpack the system of working life relations with an explicit public health perspective seldom used in more mono-disciplinary studies. 

## 3. Results

Although the Nordic countries have many important differences, they also share many common features, which may justify calling their model “the Nordic model”. The Nordic countries often figure among the world’s leading countries in various international rankings of prosperity, productivity, competitiveness, and technological modernization [15]. In addition, the Nordic countries are at the top in measurements of softer variables such as social equality, trust, life satisfaction, happiness, gender equality, social mobility, work satisfaction, and so on [16]. Stated differently, the Nordic countries seem to have managed to combine a high degree of welfare and social protection with economic efficiency and wealth creation.

Table 1 presents a selection of economic and social indicators related to working life relations, and that will be further discussed in this article. Table 1 includes the Nordic countries as well as a selection of other European countries and the US. It shows that the Nordic countries are wealthy. Except for the US, the Nordic countries as a group have the highest income (gross domestic product/GDP) per capita. However, the Nordic countries distribute their income very differently compared to the US. Table 1 shows that the Nordic countries have the lowest wage inequality of all countries, and also the lowest poverty rate (as a group). These results are closely related to the high union density rates in the Nordics (see the discussion below). The Nordic countries also have very high levels of social trust compared to the other countries covered in Table 1. Life and work satisfaction are also high, while the extent of temporary work is higher in the Nordic countries than in both the UK and the US, but lower than in other European countries.

The system of labor relations constitutes an important part of the Nordic societal model, and the way the labor market is organized has been crucial for developing the rather unique combination of good economic performance, low income inequality, and good work-related health in the Nordic countries. We use Norway as a Nordic case to defend and explain this claim, before we turn to the question of how globalization and new technology may affect Norway’s system of labor relations.

### 3.1. The Nordic Working Life Model: A Brief Overview

#### 3.1.1. Macro Perspectives

Both employers and employees are well organized in Norway (and the other Nordic countries), and this has laid the foundation for relatively centralized wage bargaining and close collaboration at the national level between a strong trade union movement and centralized employers’ associations. Trade union participation is about 80% in Iceland and close to 70% in Denmark, Finland, and Sweden. Norway has trade union participation of about 55%, and the participation in the employers’ association in the private sector in Norway is about 60–65%. 

The relations between the labor market parties in Norway have been developed over many decades. In Norway, an important early step towards coordinated and centralized wage bargaining was taken with the signing of the Basic Agreement between the Norwegian Confederation of Trade Unions and the Norwegian Employers’ Confederation in 1935. The Basic Agreement laid down formal rules and codes of conduct for collective bargaining and outlined the principles for handling conflicts of interest between employers and unions at both the national and workplace levels. There is broad agreement that coordinated wage bargaining and the close collaboration between labor and capital that emerged between the First and Second World Wars has been essential for developing Norway’s working life model [17,18,19,20]. Many of the central features often associated with the Nordic model, such as low unemployment, high productivity, and small wage differentials, would have been very difficult to achieve without a system of coordinated and centralized wage setting.

Norway replaced bargaining at the industry level with wage negotiations between national associations of unions and employers in the 1950s. As white-collar union associations united the centralized negotiations, the coverage of the central agreement spread out to almost the whole economy [20]. One particularly important effect of this was that it enabled unions and employers in export-oriented industries to establish the rate of wage increase for other groups in the economy (those working in industries with a more domestic market orientation in particular). The idea that the unions representing workers in export-oriented industries should set the norm for what unions can demand in other industries was formally implemented in Norway during the early 1960s and has been a key aspect of the wage bargaining system ever since. This has clearly helped in securing strong competitiveness and low unemployment [21,22,23]. Another important effect was that centralized wage bargaining stimulated modernization and productivity growth by forcing low-productive companies out of business and stimulating the growth of highly productive companies [18]. In a decentralized bargaining system, wages vary according to the productivity of the specific company. In a centralized system, wages are set according to the average productivity of the industry as a whole. Centralized wage bargaining thus forces wages up in low-productive companies and keeps wages down in highly productive companies. Reduced profits in low-productive companies and increased profits in highly productive companies have led to the reallocation of labor and capital from low-productive to highly productive activities over time.

In parallel, centralized wage bargaining enabled the unions to implement an ambitious egalitarian wage policy. “Solidaristic wage bargaining”, as the policy was named [18], aimed at raising the wages of the lowest paid by holding down the wages of the highest paid. This type of wage compression clearly required centralized coordination. Similar to the other Nordic countries, Norway’s trade union movement has always been shaped by an ideal of equality, especially the demand for equal pay for equal work. Trade unions thus try to contribute to reducing wage inequality whenever they can influence the determination of wages [24]. When wage negotiations are carried out at the company level, the trade unions compress wages among the employees of each company. When wage negotiations are carried out at the sector level, the trade unions compress wages among the employees of different companies within the same sector. When wage negotiations are carried out at the national level, the trade unions compress wages between companies, sectors, and occupational groups. Over the years, the policy of solidaristic wage bargaining has generated a highly egalitarian distribution of wages in Norway [25].

#### 3.1.2. Micro Perspectives

For workers, collective bargaining not only delivers a fair share of technology and productivity growth but also provides voice and empowerment. For employers, collective bargaining resolves disputes and legitimizes management control through agreed procedures. Norway’s system of labor relations therefore seems to gather broad support among both employers and trade unions [17].

The strong ties between national labor market organizations in Norway have their counterpart within companies. The centralized bargaining system is complemented by well-developed workplace structures for negotiations, co-determination, and local wage adjustments. At the company level, these structures ensure the interests of both employers and employees and they enable employees to be consulted on issues related to working conditions, health and safety, and the development of the company. Most large companies and public sector organizations in Norway have formal arrangements (councils), comprising employee and employer representatives, to ensure co-determination and involvement [17]. For example, the Basic Agreement (see above) states that: “Matters that are of material importance for the employees and their working conditions, which relate to the activities of the enterprise, substantial investments, changes in systems and methods of production, quality, product development, plans for expansions, reductions or restructuring, shall be submitted to the council for its opinion before any decision is made.” (quoted from Løken et al. 2013 p. 50 [17]). Norway’s Working Environment Act makes working environment committees compulsory in all large companies (>50 employees), but many smaller enterprises choose to have similar arrangements. Their aim is to preserve the interests of employees in matters relating to the working environment, both physical and psychosocial.

#### 3.1.3. Trust Binds Macro and Micro Together

The level of social trust is higher in the Nordic countries than other countries. The results from the World Values Survey show that Norway had the highest trust score among all countries [26], with 74% classified as trusting other people. The other Nordic countries are also high on the list (Table 1). 

It is well documented that trust is closely correlated with income inequality [27,28]. Countries with low income inequality tend to have higher levels of social trust. In Norway, there are strong reasons to believe that trust is both a cause and a consequence of equality. At the macro level, the high level of trust between the labor market parties has been crucial for developing and maintaining coordinated and centralized wage bargaining. Coordinated and centralized wage bargaining has contributed to low wage inequality through solidaristic wage bargaining, which again has contributed to high social trust. Further, high social trust has created stronger political support for various types of redistribution policies funded by taxes [29]. The equality created by solidaristic wage bargaining and redistributive welfare policies has contributed to even higher levels of trust, and this has enabled income inequality to be further reduced through solidaristic wage bargaining and an expansion of the welfare state, and so on. Over the long term, this feedback process has created a situation in which Norway has ended up with low wage inequality, a strong welfare state, and high social trust.

At the micro level, social trust has contributed to high wealth creation through more efficient organization of enterprises, without too much hierarchy, administration, or surveillance.

#### 3.1.4. Democratic Innovation

Small differences between employers/managers and employees, and a high level of employee involvement in decision-making processes at the company level, characterize the Norwegian working life model. Seen in this way, the Norwegian system for working life relations can also be viewed as an important part of a larger system for democratic innovation. Norway’s workforce has high formal and informal qualifications, and it is therefore important to facilitate the efficient utilization of these resources. High levels of social trust help with this. When employers trust their employees, a social foundation is established that enables employees to take initiative, use their own problem-solving abilities, and be actively involved in restructuring processes and the development of the company. Furthermore, workers at the operative level (“the shop-floor”) often know best where “the shoe pinches” and where innovations and new ways of solving problems are most needed. Worker participation is probably particularly important for the small and incremental improvement of work methods, organization, and products. In the European Working Condition Survey [13], 82% of the workers in the Nordic countries report that they have the power to choose or change their methods of work, while 57% report that they can influence decisions that are important for their work, including health and safety activities. The corresponding figures for the other countries covered by the survey are much lower. 

The combination of trust, decentralized decision-making, and worker involvement is probably a major explanation for the high productivity and wealth creation in Norway’s enterprises [30]. In addition, high wealth creation contributes to a generally high level of well-being in the population, since the pie that is produced is distributed in a reasonably fair manner through a well-developed system for collective wage bargaining.

Trust also promotes democratic innovation in other ways, including the fact that trust plays an important role in cooperation and the coupling of resources and ideas. Where there is trust, there is less need for protecting one’s own interest through detailed agreements and contracts. Transaction costs are reduced, and it is easier for actors to initiate relationships and secure joint gains. Trust also makes people more willing to embrace new ideas and to try new ways of doing things, and it lowers the threshold for making innovation happen. When people trust others, they feel secure enough to take risk and allow themselves to be vulnerable. As Botsman [31] shows, trust acts as a bridge between the known and the unknown, thereby helping people to take “trust leaps”. Such trust leaps occur when people take a risk to do something new or to do it differently from the way that they have done something before.

### 3.2. Employment, Working Environment, and Health

**Figure 1:**
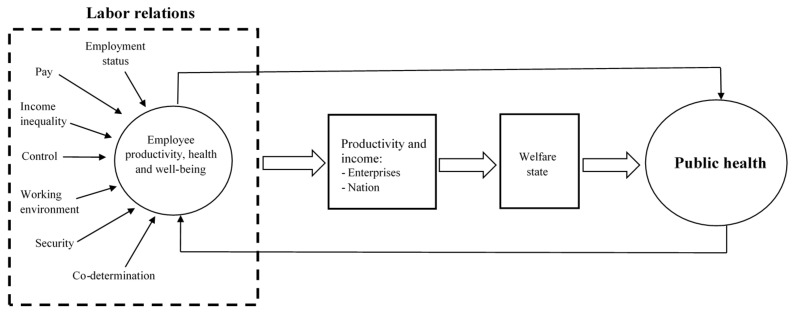
Relationships between labor relations and public health.

As already noted, work is generally regarded as positive for health [32,33]. Nevertheless, a poor working environment may result in sickness and disease, whereas a good working environment may result in well-being and flourishing. Several studies have shown that the working environment is more important for social inequality in health than lifestyle factors [34,35,36,37]. Occupational health promotion is therefore not only important for the individual employee but also for the entire society. Figure 1 shows factors related to the labor market (such as employment status, pay, working environment, and co-determination) that are important for occupational health and well-being, and that healthy and creative workers are important for productivity. High productivity secures incomes for the welfare state through taxes. The welfare state provides various services for the population, which again feeds back into individual and public health. A healthy population is also expected to positively affect the labor market.

Based on the Sixth European Working Conditions Survey, we present some facts about the labor force and working environment in Norway compared with that of the European Union (EU). The survey includes 35 European countries and 44,000 employed people [13,38].

*Employment*. Among those 20–64 years old in Norway, 79% (81% for men and 77% for women) are employed or self-employed. Only Sweden and Switzerland have higher employment rates, and no other country has a smaller difference between men and women.

*Working hours and job–family interaction*. During the past 25 years, working hours have declined in both Norway and the EU, whereas part-time work has increased. In 2015, only three countries had a shorter average working week than Norway. Norway is the country in Europe where the most employed people (45%) report that they have the opportunity to influence working hours (EU: 18%). The proportion in Norway considering the working hours to be good or very good in relation to family and social obligations is also the highest in Europe (87%).

*Working environment*. In both the EU and Norway, most employed people are overall satisfied with their work, but in no other country are more workers satisfied than in Norway (96%) (EU: 86%).

The mechanical, physical, chemical, and biological working environment is relatively good in Norway, although the proportion of workers doing heavy lifting and who are exposed to biological risk factors is the same as in the other European countries. The figures for quantitative and emotional work demands are somewhat difficult to interpret, but more employees in Norway than the EU average state that they work at a high pace, that they are often disturbed at work, and that they are controlled by work others have done or by direct claims and requests from others.

Based on these results, it does not appear that the physical, chemical, or quantitative work demands make employees in Norway more satisfied with their working conditions than others. Employees in Norway score especially high on decision authority: The opportunity to decide or change the order of work tasks, working methods, and pace of work. In addition to being able to determine their own working hours, many reported that they have a secure job (about 90%), participate in development processes at work, and receive training for the work paid by the employer. Employees in Norway also experience very high support from colleagues (88%, the highest in Europe) and also from the nearest manager (75%).

According to the well-known demands–control–support model [39], high work demands (amount of work, pace, etc.) harm creativity, productivity, and health. The same applies to low control (self-determination) and low social support (poor colleague relationships). What is important according to this model is the combination of demands and control or social support. This means that high job demands especially harm health and well-being if decision authority or social support is also low. The negative health effect of high job demands is significantly reduced if employees have good opportunities to decide for themselves how to perform the tasks or have good support from colleagues and management. This is called the stress hypothesis. The model’s second hypothesis, the learning hypothesis, argues that the combination of high work demands and a high degree of decision authority or social support actually benefits learning, mastery, and development—which in turn contributes to engaged, creative, and productive workers. Norway is known for having high productivity, and this can partly be explained by the fact that employees in Norway have high overall work demands combined with a high degree of control at both the individual and collective levels [40].

*Health.* With such a point of departure, employed people in Norway would be expected to have relatively good (work-related) health, and they do. Nevertheless, Norway has the highest sickness absence in Europe and the same prevalence of musculoskeletal symptoms, injuries, and fatigue as Europe. Although the World Health Organization [41] defines health as not merely the absence of disease, most studies on health focus on the prevalence of disease. The positive parts of health, such as mental well-being, coping, learning, and engagement, are often neglected [41,42] and are also often closely related to work [43,44]. For such factors, employees in Norway score particularly high on mental well-being, job engagement, and learning new things at work. In addition, Norway has the highest proportion of workers in Europe saying that the job contributes positively to their health (30%). There are reasons to believe that these issues are closely associated with the fact that employed people in Norway are more satisfied with their work than in any other European country.

In a study including nine European countries (Sweden, Finland, Spain, Portugal, Hungary, Bulgaria, the Netherlands, Germany, and the United Kingdom) representing different societal models, Drobnič, Beham, and Präg [43] showed that employees in the Nordic countries score highest on both job satisfaction and life satisfaction (see also Table 1). Of the many work-related factors influencing both the job and life in general, one factor is especially important: A secure job. Thereafter follow good wages, high autonomy at work, and interesting work tasks, although any association between high decision authority and interesting work tasks and quality of life is mediated by job satisfaction, secure jobs, and good wages directly affect the quality of life [43].

Since the 1970s, the Whitehall studies have shown that work, and especially the resulting socioeconomic status and psychosocial working environment, is important for health and social inequity in health [45,46,47]. The importance of work contracts and how working life is organized is less frequently investigated. Nevertheless, studies show that workers with atypical or non-standard work contracts have higher mortality [48] and poorer physical and mental health [49,50] than workers with typical or standard work.

A review study shows that 7 of 13 studies documented that workers with atypical working contracts had a higher risk of work-related injuries and accidents than employees with standard working contracts [51]. The reasons for such differences in health are not frequently investigated. In a summary of the consequences of atypical working contracts, Howard [52] suggests that workers with atypical contracts often have to perform the most dangerous and demanding work. In addition, they have less personal protective equipment available, do not get good enough training for the work and for health and safety, and have less opportunity to refuse to perform dangerous and demanding work. In addition to these factors, atypical contracts would be expected to lead to a low sense of belonging to the workplace, low social support from supervisors and colleagues, and low social security and trust in general. All these factors are associated with poor health [53,54].

### 3.3. How Globalization Affects Labor Markets

Based on the review of literature presented above, one can conclude that Norway and the other Nordic countries are doing rather well when it comes to productivity, equality, working environment, trust, and health and well-being, and that these factors are related to how working life is organized. In the following sections we will focus on trends related to globalization and technology that may put the Nordic working life model under pressure and thereby threaten future public health and well-being.

#### 3.3.1. Migration

Norway has received relatively many refugees from countries in Africa, Asia, and the Middle East in recent years. These refugees have often low occupational and linguistic skills that result in problems in entering Norway’s labor market (employment rate below 50%) [55,56]. Nevertheless, the employment rates vary greatly by country background, immigration cause, time in Norway, age, and sex. The fact that many immigrants are not working and that those in work have low wages, low levels of unionization, and often atypical forms of employment can contribute to creating an unfortunate “we versus them” dimension in society and thus also in the labor market. This may weaken the support for Norway’s working life model and possibly the welfare model as a whole [12]. Although this issue is relevant for the labor market in Norway and the Nordic countries, we regard labor migration to be more important for the labor market and how it is organized, and thereby for occupational and public health.

After several Eastern European countries joined the EU in 2004, Norway experienced higher labor migration than any other European country [57]. These workers have on average low education and are mainly employed in labor-intensive industries with relatively low productivity [58].

The effect of this immigration on Norway’s economy and Norway’s workers is an important political and economic question. Nevertheless, the knowledge base is poor, and little is known for sure. The period from 2004 to 2014 was very good for Norway’s economy. Unemployment rates were low, and income growth was high. Norway depended on labor immigration in this period to get all pending work done, and the immigrants came because well-paid jobs were available.

Hoen et al. [59] investigated how immigration affects the labor market. They found that employees with low income lose out with labor immigration, because they have to compete with low-education and low-wage foreign workers, whereas highly educated and highly paid workers have benefited from immigration. Løken et al. [60] hold, however, that there are major methodological problems associated with measuring and quantifying such effects. They interpret the results of Hoen et al. [59] differently. They argue that all people living in Norway have benefited from the immigration, but that the people with higher socioeconomic status have benefited more than those with lower socioeconomic status.

Further, labor immigration can have several positive and negative effects for the recipient country that Hoen et al. [59] did not capture. One effect may be change in how contracts and employment conditions are designed in the labor market. Such changes may in the end affect the job situation of both the native workers and the work immigrants.

#### 3.3.2. From Standard to Atypical Employment Relationships

In Norway, about 90% of all workers have standard employment, meaning a continuous bilateral employment relationship between an employer and a worker. Standard employment provides more security and greater predictability than temporary employment. New technology, internationalization of the labor market, and high labor supply challenge the system of standard employment.

In an international context, the number of employees who are working without a standard job contract and have an atypical connection to the labor market is growing [61]. This has not yet clearly happened in Norway, but it is developing in certain industries [62,63]. Standing [61] refers to the people who are (partly) working without standard employment as a new class: The precariat (combining the words precarious and proletariat). The precariat earns their living through short-term jobs or they are hired as workers, callers, or freelancers. They are often registered as self-employed, and relatively many are immigrants. Many alternate between receiving transfer payments and short-term work. Atypical employment can be an advantage for staffing companies and for groups of highly educated people with specialized expertise, in addition to students and people in a similar situation who want short-term assignments. For the group defined as the precariat, this work situation is not desired. The result is lasting insecurity and stress, with great potential for adverse health effects. The precariat differs from the proletariat in that they are not professionally organized. We present here some atypical forms of employment, further discussed in Eldring and Ørjasæter [62] and Ingelsrud et al. [63].

*Hiring by staffing agencies.* The use of hired labor through staffing agencies has increased significantly in Norway over the past two decades. Nevertheless, registered hired workers do not account for more than 1.5–2% of all person-years in employment. The proportion of employees employed by staffing agencies is probably higher than the percentage for person-years indicates, because hired employees are probably less likely to have a full-time job than permanent employees. The use of such labor is quite common in some industries and in some regions. Within building and construction, hired workers performed 10% of all artisan services in 2017 [64]. In the Oslo region, hired workers performed 16% of such services, and the proportion was as high as 35% at some large construction sites. The use of hired workers is also common in other industries such as hotels and restaurants, transport, and health and social care.

The advantage of using hired labor is that it is cheap and flexible for the companies, which can easily adjust the use of labor according to fluctuating demand for their products or services. In 2017, standard employment without guaranteed pay (also called a zero-hour contract) was the most common contract used by staffing agencies [65]. This means being registered as a permanent employee in the agency but only being paid when working for the agency’s clients. This form of “permanent” employment is likely to result in unpredictability and an unstable income. In addition, unionizing is difficult. In 2019, following a fierce battle between the right- and left-wing political parties in Norway, the Working Environment Act was changed, resulting in a ban on the use of standard employment without guaranteed pay.

Eldring and Ørjasæter [62] emphasize that the legislation governing the use of hired labor is very difficult to relate to and that many different schemes for adapting to the legislation exist. This results in the fact that many serious, but especially smaller and disreputable companies can easily break the rules, both intentionally and unintentionally. In addition, hiring labor challenges the system of ordinary employer and employee relationships in which collective bargaining largely governs income and working conditions.

*Temporary employment.* Temporary employees have been used in Norway’s labor market for a long time, and this has been an important source of conflict between employers and employees’ organizations. In 2015, the Working Environment Act was liberalized to give companies more opportunities to use temporary workers. In addition, an official white paper the following year recommended relaxing regulations on working time [66]. In principle, there are two types of temporary employees: Ordinary temporary staff and temporary substitutes. The first type involves being employed for a limited period of time but having the same working conditions and rights as those with permanent employment. This is probably the best form of temporary work but usually results in having an insecure working situation and income. This form of employment is common in academia in connection with time-limited project work. Temporary substitutes are especially common in the hotel and restaurant industry and in health and social care services. The employment contract for these workers is very similar to permanent employment without guaranteed pay, which has been used by staffing companies (see above).

Many who have their main occupation elsewhere, such as students, are often happy to be on-call to be able to earn some extra money without committing to an employer–employee relationship. Temporary employment gives the employer flexibility in planning operations, but in Norway, it is only legal if the employer has no other way to meet the need for labor. Using temporary substitutes to meet a permanent need and thus not a need that suddenly occurs is illegal. Interpreting “permanent need” is often difficult.

An employee who has a contract for temporary work does not have the same rights as a permanent employee and therefore does not have the right to sick pay or any protection from dismissal should there be any conflict with the employer. In addition, these employees have difficulty in dealing with questionable issues related to the operation of the business and health and safety.

*Employed by a foreign company.* Posted workers are another group that often work on short-term contracts. EU (and EEA) regulations include not only the free movement of people between countries but also of services across borders. The posted workers work in Norway but have their employment contract in a company outside Norway, most often Eastern Europe. The company may be a staffing company but could also be other companies with or without standard working contracts, or hardly any employment contract whatsoever.

In the early 2000s, these workers had wages and working conditions similar to the conditions common in the country from which they came. Now the disputed EU posting of workers directive states that the undertaking (such as in Norway) should ensure that the employer of the posted workers (such as in Eastern Europe) follows the laws and regulations governing working hours, wages, and health and safety in the country in which the work is performed [67]. This seems to be a good arrangement to prevent social dumping. In practice, it has been proven that enforcing this directive is difficult, and the worst examples of social dumping in Western Europe have involved posted workers [68].

The use of posted workers challenges Norwegian working life and Norway’s working life model since this group of workers often works under poor conditions and with lower wages than other workers in Norway’s labor market. In addition, they are not part of the collective wage bargaining system and thus disrupt the conditions for competition between both Norwegian and foreign companies.

*Self-employed people.* Closely linked to precarious work is the use of self-employed people. Self-employed people include those who do not have an employer. The proportion of self-employed people in Norway is about 7%, which is the lowest in Europe (EU average: 15%) [69]. Self-employed people are very heterogeneous—from those with no/low education and low income (such as farmers, fishers, drivers, artisans, and artists) to those with high education and income (such as lawyers and physicians) [70,71]. By far the largest group of self-employed people have no employees. In general, self-employed people have poorer occupational safety since Norway’s Working Environment Act does not apply to them. In addition, they have poorer conditions regarding available welfare benefits, such as sickness and unemployment benefits. In Standing’s [61] conceptual use, poorly paid self-employed people and freelancers are defined as the precariat.

Employing self-employed people can be advantageous for employers because they do not have to deal with collective bargained wages and minimum wage rates, and they can bypass working time rules and avoid paying sick pay. Legislation in Norway imposes some restrictions, but there are unclear rules with possibilities for “creative” and more or less legal solutions. Eldring and Ørjasæter [62] describe this group of self-employed people as “masked employees”. That is, they work as regular employees but without having the same rights as permanent employees. Increased use of self-employed people or freelancers will result in more workers with (apparently) more decision authority but who live more insecure lives in finances and social security.

### 3.4. How New Technology Affects Labor Relations

#### 3.4.1. The Platform Economy

The extent of the “platform economy” or “sharing economy” has risen as a result of developments in information and communication technology [72,73]. Well-known examples of platform services are Airbnb (hire of accommodation) and Uber (taxi rental). What is special about this is that, in addition to the client (customer) and provider (employed), a technology supplier provides the technological platform through which the work is communicated. The transaction is between the customer and the employee, but the transaction cannot be carried out without a technology provider that mediates the contact between the two parties to the transaction. This intermediary is by definition not an employer. The worker is often self-employed, a freelancer, or the like. Getting an overview of this part of the labor market is difficult because there are so many forms of technological solutions and forms of connections, and because it is rapidly developing. Some platforms are local (such as Uber) and others are not (such as low-cost information technology services). Although some platform owners only develop the technology and make it available, others are more actively involved in the employment and pricing process itself.

Although an estimated 10,000 to 30,000 people in Norway used a sharing platform in 2016 and 2017, a survey of the platform economy in Norway concludes that it is currently relatively marginally developed [74]. Nevertheless, a Norwegian white paper [72] concludes that the platform economy has the potential to change how Norway’s working life is organized and thus also the conditions that regulate it.

#### 3.4.2. Automation and Digitalization

According to Statistics Norway, as many as 33% of all jobs in Norway are likely to disappear over the next 20 years as a result of new technology and the automation of work tasks. Ever since the Industrial Revolution, there has been a fear that machinery and new technology will replace human labor. But this has not happened [75]. On the contrary, new technology has led to increased productivity, value creation, and prosperity. Labor freed up as a result of automation and modernization has been transferred to other parts of the economy that have expanded.

Nevertheless, dismissing the concern many people have for what may be the consequences of the ongoing technological change is difficult. One reason is that technological change is going much faster today than before. The pace of restructuring in society is accelerating, and many people may therefore lose their jobs as a result of restructuring. This is a challenge for those who have difficulty finding a new job after a job loss, such as older workers or workers with little formal education. In addition, more advanced technology makes working life more demanding. Compared with before, there are far fewer jobs today for those with low qualifications and education.

Globalization and weaker trade unions enhance these problems. In addition, labor’s share of total income is now falling in many countries [4]. Income going to capital owners is increasing, and these incomes are increasingly being used for investing in low-cost countries. Investment in more advanced countries (including Norway) falls, and fewer new jobs are created there. In addition, lower increases in wages contribute to lower demand for goods and services. As the growth in consumption of goods and services flattens, production and employment will also grow less. Those with high incomes typically save a larger share of their income: That is, allocating more to affluent people reinforces the problem of declining demand and thus lower job creation. Thus, the trend seems to be that new technology, automation, and digitalization increase productivity and income (to capital owners) but remove jobs. Further, the growth in job creation in other parts of the economy is too weak to compensate for job losses resulting from new technology.

Future employment appears to grow in industries in which the proportion of workers organized in trade unions is low [57]. This can lead to further downward pressure on wages, greater income disparities, and a strengthening of the processes described above.

### 3.5. Changing Management Culture

An increasingly more globalized labor market is expected to accelerate greater diversity in the corporate and management culture. In Norway, drivers in this context may be more foreign business owners, managers from other cultures, employees who are used to different types of management than what is common in Norway and Norwegian leaders with education from abroad who bring with them management ideas from other cultures. This can undoubtedly contribute positively to diversity, better management, higher productivity, and competitiveness, and much learning in Norwegian working life. At the same time, it can also undermine the system of labor relations that has been developed in Norway over decades—which is based on a humanistic view characterized by power balance, equity, co-determination, and a consensus-oriented model of decision-making [76]. Some argue that human resource management in Norwegian working life is increasingly becoming Americanized by relying on management principles derived from a more individualistic labor market. This may end up putting pressure on Norway’s working life model [40,76,77,78].

Norway’s working life model recognizes that employers and employees have conflicting interests. This is an important reason why, also in the form of legislation, schemes have been introduced in Norway that ensure employee influence through collective representation in corporate boards, cooperation committees, and working environment committees at the company level. At a higher level, employers and employees’ organizations closely communicate with the authorities about matters concerning the development of Norway’s labor market, such as the national agreement on creating an inclusive working life [79]. In a more liberalist tradition, participation is not as much in focus and the proportions of organized employers and employees are often low [7]. In addition, wage bargaining is more decentralized, which enables greater wage differentials and status differences. Outsourcing of tasks is common, and short-term contracts, part-time employees, and hired labor are often used [7]. Scorecard management, often broken down to the individual level, is a key part of the company’s daily operations [77]. Such management is often associated with private companies but has also affected public-sector enterprises in many countries, including Norway [80]. This is often referred to as new public management.

The annual survey on co-determination in Norway’s labor market [40,78] identifies changes in work, work organization, and management. It shows that the formal schemes related to corporate democracy (such as collective bargaining agreements, trade union and health and safety representatives, working environment committees, and employee representatives in boards of directors) are well established in both Norwegian companies and foreign companies operating in Norway. This pattern changed slightly from 2009 to 2016. This supports a claim that Norway’s working life model and Norwegian corporate democracy are strong and have support among employees, trade union representatives, and managers. Nevertheless, compared with 2009, significantly fewer employees, trade union representatives, and managers in 2016 felt that they had great influence on their own work and their own workplace. This applies to how they experience influence on their own work situation (individual level), influence on the work organization (group level), and influence on the management and organization of the business (organizational level) [40]. In accordance with this, the survey also shows that as many as 45% of the respondents in 2016 think that working life is moving in a more authoritarian direction, and only 10% think that working life is moving in a more democratic direction. Employees in foreign-owned enterprises report less influence than employees in Norwegian-owned companies. It is argued that “imported” management systems can easily be adapted to Norway’s model of participation and co-determination, but the researchers behind the co-determination survey state that their findings do not support such a claim [40].

Norway’s Working Environment Act is relatively strict and guarantees workers’ rights, but how companies comply with these rights and how they permeate business cultures varies widely between businesses and between industries. It will be crucial for Norway’s working life model for managers to recognize the importance of having unionized workers with collective representation in the management of the companies. If Norwegian personnel and working life management are further Americanized, one can assume that a good working environment strongly emphasizing co-determination will deteriorate and that Norway’s overall working life model will be undermined.

## 4. Discussion

### 4.1. Relationships between Globalization, the Nordic Working Life Model, Inequality, Trust, and Public Health

**Figure 2:**
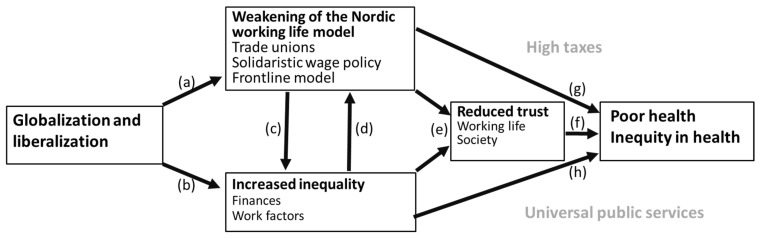
Relationships between globalization, the Nordic working life model, trust, and public health.

Norway’s system of labor relations functions well, with extensive collaboration between employees and employers at both the national and company levels. The working conditions are generally good, and employees report that they generally thrive at work and are in good health. However, increasing globalization and technological change challenge this situation. Conditions related to social life and health are closely related to how working life is organized. Figure 2 summarizes the factors and contexts presented above that we think are key to public health in Norway and in the other Nordic countries. Although the factors largely influence each other, the arrows illustrate which direction we think is most important. The gray text represents societal conditions that influence all the previously mentioned factors and their relationship, but are not mentioned here because they are not inherently related to working life.

Globalization and technological change associated with liberalization of the labor market can lead to social inequalities in terms of income, employment, working conditions, and working environment (Figure 2a). Further, the Nordic working life model (Figure 2b), which is based on coordinated wage bargaining at the national level and the bargaining system of letting the companies exposed to international competition setting the framework for the negotiations in other sectors, is weakening. In addition, and most importantly, the model recognizes that employers and employees are unequal in power and that employees, through trade union representatives, therefore should play an important role in managing industry.

Both employers and employees have traditionally benefited from low income inequality [81], but possible weakening of the working life model through, for example, low levels of organization among employers and employees, especially in the sector exposed to international competition, may lead to increased social inequality (Figure 2c). Further, increased social inequality caused by globalization and technological change weakens the working life model because the weakest workers (the precariat) do not see that they may benefit from being unionized themselves and thus be part of the coordinated wage bargaining that is so crucial to the Nordic working life model (Figure 2d). Both the Nordic labor market and the Nordic countries as a whole have high levels of generalized trust. This is likely to be weakened (Figure 2e) because of increased social inequalities and reduced employee co-determination in the labor market. Trust and security are important factors for good health, and diminished trust may therefore weaken occupational and public health (Figure 2f). In addition, increased income inequality and inequality in working conditions and working environment (high demands and low control and social support) cause social inequalities in health and directly and adversely affect the average health of workers and the population (Figure 2g). Weakening of the Nordic working life model may also directly affect occupational and public health (Figure 2h).

### 4.2. Further Research 

The current article has described and discussed trends in the labor market related to globalization and technological changes and which consequences these trends may have on social inequality, trust, well-being, and public health in the future. There is a need for empirical research investigating whether the trends and associated changes will persist, and whether these changes will have an impact on health and well-being as suggested by us. In addition to longitudinal studies investigating the trends and their effects, we call for (natural) experimental research, and studies making use of participatory action research methods seem particularly relevant.

So far, the literature regarding democratic innovation (e.g., [82,83]) has mainly focused on how citizen participation can be achieved when it comes to influencing political decisions and public governance. We argue that there is an urgent need for more focus on how the labor market in general, and companies in particular, are governed and how democratic innovations may be promoted in this sector of society. It is then important to focus on both worker participation through representatives (e.g., labor unions) and through engaging individual workers outside traditional organizations or institutions. We have shown how changes in information and communication technology may challenge the Nordic working life model and a relatively egalitarian labor market (and society) with a high degree of trust between parties. Nevertheless, we believe that such new technology may be highly relevant to promote worker participation in traditional “physical” companies, but particularly for workers employed within the platform economy since such workers often work independently of others and are rarely united in efforts to improve working conditions and income. To our knowledge, research on this topic is so far negligible. Whereas we have focused on how globalization and technological change may threat the Nordic working life model, another and perhaps equally challenging threat to the Nordic working life model is related to changes that have taken place inside the Nordic countries in past decades. Due to the Nordic working life model with its alliance between employers and unions for low paid workers, the social mobility has increased, and the middle class has therefore grown to constitute the majority in society. An emerging question is whether the growing middle class will continue to support a solidaristic wage policy and wage compression. After all, this is a wage policy that makes people of the middle class lose out in an individualistic sense. Together with challenges related to globalization, we think that this internal challenge to the Nordic working life model deserves attention from both politicians and researchers in the future.

## 5. Conclusions

The Nordic working life model has contributed in creating an egalitarian society with low income inequality, high social trust, and good public health. The model has previously proven to be strong in the face of international challenges. Nordic companies are competitive, and Nordic employees are resourceful and adaptable, have a high level of expertise, and thrive at work. These factors should make the Nordic companies and the Nordic societies at large better equipped to meet the challenges of the future than most other countries. Nevertheless, the challenges related to new technology and globalization arising today and discussed in this article may differ from challenges in the past. We emphasize that we have not yet seen a grave negative impact of globalization on the Nordic labor market, but if social inequalities continue to increase, we will in the future without doubt see more social and political conflicts. Therefore, politicians and other stakeholders need to address and tackle the development of social inequalities more efficiently than before. Combined with traditional national policy measures, one option may be to develop and try out democratic innovations going beyond familiar institutionalized forms of worker participation. 

## Figures and Tables

**Table 1:** Selected measures of economic and social indicators broadly related to working life outcomes.

Countries	GDP Per Capita ^1^	Union Density ^2^	Bargaining Coverage ^3^	Wage Inequality ^4^	Poverty Rate ^5^	Trust ^6^	Temporary Employment ^7^	Work Satisfaction ^8^	Life Satisfaction ^9^
Nordic countries	55,842	66.7	85.2	2.5	7.0	65.0	11.6	36.3	9.6
Denmark	55,138	66.5	82.0	2.6	5.5	64.5	10.9	47.0	9.7
Finland	48,248	60.3	89.3	2.6	6.3	58.0	15.6	28.0	10.0
Iceland	57,453	91.8	92.0	2.9	5.4		7.8		9.5
Norway	65,603	49.2	72.5	2.5	8.4	73.7	8.0	44.0	9.9
Sweden	52,766	65.6	90.0	2.1	9.3	63.8	16.6	26.0	8.9
Continental Europe	52,240	23.7	85.4	3.0	9.3	313	13.7	30.4	7.8
Austria	55,529	26.3	98.0	3.1	9.8		8.7	41.0	8.3
Belgium	50,442	50.3	96.0	2.5	9.7		10.9	29.0	7.6
France	45,149	8.8	98.5	3.0	8.3	18.7	16.4	21.0	6.1
Germany	53,752	16.5	56.0	3.3	10.4	32.1	12.0	30.0	7.8
Netherlands	56,326	16.4	78.6	3.0	8.3	43.0	20.3	31.0	9.3
Southern Europe	35,990	20.9	65.8	3.1	14.0	23.7	19.1	19.1	3.6
Greece	29,592	20.2	25.5	3.2	14.4		12.5	23.0	2.2
Italy	41,426	34.3	80.0	2.3	13.7	28.3	16.7	18.0	4.4
Portugal	33,035	15.3	73.9	3.6	12.5		20.8	18.0	2.4
Spain	39,908	13.6	83.6	3.1	15.5	19.0	26.3		5.5
UK	45,505	23.4	26.3	3.4	11.1	30.0	5.2	37.0	7.2
USA	62,480	10.1	11.5	5.0	17.8	38.2	4.0		7.4

^1^ Gross domestic product (GDP) per capita in US dollar, converted to international dollars using purchasing power parity rates, 2018. Source: OECDi*Library*. ^2^ Trade union density corresponds to the ratio of wage and salary earners that are trade union members, divided by the total number of wage and salary earners, 2018. Source: OECDi*Library*. ^3^ Collective bargaining coverage rate expresses the number of workers covered by one or more collective agreements as a percentage of the total number of workers, 2016. Source: OECD.*Stat*. ^4^ Dispersion of pre-tax hourly wages measured by the 90–10 ratio. The ratio shows the income level of individuals at the top of the income distribution (top 10%) relatively to the income level of those at the bottom of the distribution (bottom 10%), 2018. Source: OECD Earnings Distribution Database. ^5^ The number of people (in percent) whose income falls below half the median household income of the total population, 2017. Source: OECD.*Stat*. ^6^ Share of people agreeing with the statement "most people can be trusted”, 2009–2014. Source: World Value Survey. ^7^ Temporary employment includes workers whose job has a pre-determined termination date. Source: OECD.*Stat*. ^8^ Number of respondents who answer that they are “very satisfied with working conditions in my main paid job”, 2015. Source: Sixth European Working Conditions Survey. ^9^ Life satisfaction measures how people evaluate their life as a whole. Source: OECD Better Life Index.
